# Microwave dielectric characterisation of 3D-printed BaTiO_3_/ABS polymer composites

**DOI:** 10.1038/srep22714

**Published:** 2016-03-04

**Authors:** F. Castles, D. Isakov, A. Lui, Q. Lei, C. E. J. Dancer, Y. Wang, J. M. Janurudin, S. C. Speller, C. R. M. Grovenor, P. S. Grant

**Affiliations:** 1 Department of Materials, University of Oxford, Parks Road, Oxford OX1 3PH, United Kingdom; 2 International Institute for Nanocomposites Manufacturing (IINM), Warwick Manufacturing Group, University of Warwick, Coventry, CV4 7AL, United Kingdom

## Abstract

3D printing is used extensively in product prototyping and continues to emerge as a viable option for the direct manufacture of final parts. It is known that dielectric materials with relatively high real permittivity—which are required in important technology sectors such as electronics and communications—may be 3D printed using a variety of techniques. Among these, the fused deposition of polymer composites is particularly straightforward but the range of dielectric permittivities available through commercial feedstock materials is limited. Here we report on the fabrication of a series of composites composed of various loadings of BaTiO_3_ microparticles in the polymer acrylonitrile butadiene styrene (ABS), which may be used with a commercial desktop 3D printer to produce printed parts containing user-defined regions with high permittivity. The microwave dielectric properties of printed parts with BaTiO_3_ loadings up to 70 wt% were characterised using a 15 GHz split post dielectric resonator and had real relative permittivities in the range 2.6–8.7 and loss tangents in the range 0.005–0.027. Permittivities were reproducible over the entire process, and matched those of bulk unprinted materials, to within ~1%, suggesting that the technique may be employed as a viable manufacturing process for dielectric composites.

3D printing allows objects with complex geometries to be created without the need for expensive tooling, making it an attractive fabrication technique for both scientific research and commercial manufacturing. Historically, 3D printing has been predominantly employed as a rapid prototyping technique, but its use for the production of final parts continues to expand and now represents over 40% of total additive manufacturing product and service revenues^1^. This expansion relies on the development of feedstock materials which have the requisite properties for the final part yet are compatible with the printing process.

Dielectric materials with high real permittivities are used extensively in a number of important scientific research areas and technology sectors, such as photonic crystals^2^, electronics^3^,^4^, and communications^5^. Of widespread use are ceramic/polymer composite materials, composed of ceramic particles in a polymer matrix, which combine the high real permittivities of ceramics with the mechanical properties and processability (extrusion, injection moulding, etc.) of polymers. It has previously been shown that ceramic/polymer composites may be printed using a number of the sub-processes that fall under the umbrella term of 3D printing—for example ceramic stereolithography^6^,^7^,^8^,^9^,^10^ and the fused deposition of ceramic/polymer composites^11^,^12^,^13^,^14^,^15^,^16^,^17^,^18^,^19^,^20^,^21^,^22^,^23^—and that such processes may be exploited to fabricate devices that require high permittivity materials. We note here that in many of these examples the polymer is subsequently removed by burning and the ceramic sintered, whereas in other cases the polymer composite itself is used as the desired functional material—the latter approach is the focus of this paper.

The fused deposition of polymers (i.e., the technique trademarked as fused deposition modelling, and also referred to as fused filament fabrication) is the most widely used 3D printing process; it is particularly appealing in the respect that the machines are relatively inexpensive and easy to operate^1^. Fused deposition is typically used to print conventional polymers but it may also be readily used to print polymer composites containing solid particles. This possibility was noted by Crump in the original patent on the fused deposition modelling technique^24^, and a wide range of composite feedstock materials for use with fused deposition 3D printers have since become commercially available which contain, for example, metal, wood, glass, and various forms of carbon. Moulart *et al.*^16^ have reported the fabrication and dielectric characterisation of ceramic/polymer composite filament composed of BaTiO_3_ microparticles in acrylonitrile butadiene styrene (ABS) with a view to the fused deposition of electronic and microwave devices (though the ability to actually print this BaTiO_3_/ABS filament was not demonstrated in ref. 16). Preliminary results on the fused deposition of BaTiO_3_/ABS composites for electromagnetic applications have previously been presented in refs 20,21, those for a ferrite/ABS system in ref. 25, and the application of the technique for the fabrication of metamaterial structures in ref. 26. The development of ceramic/polymer filament composed of TiO_2_ in an unspecified polymeric matrix, together with the dielectric characterisation of 3D printed parts and the demonstration of their utility in a channel emulator device, has been reported in refs 22,23.

Herein we report the results of a detailed study on the microwave dielectric properties of printed ceramic/polymer composite parts with high *ε*′ values fabricated via fused deposition. We define a ‘high’ value of *ε*′ as one that is greater than the values of conventional polymers, which are *ε*′ ≈ 2–3 at microwave frequencies^27^,^28^. We used ABS as the polymer matrix because it is a standard material for desktop fused deposition 3D printers, and BaTiO_3_ as the ceramic filler because it has an unusually high *ε*′. Whereas previous work has reported essentially one-off, demonstrator, properties, we focus on: (1) explicitly demonstrating that relatively complex objects with high *ε*′ values can be 3D printed via fused deposition, (2) providing the detailed dielectric measurements with low uncertainties (~1%) necessary as input parameters into the design of real devices, and (3) comparing the dielectric properties of the printed parts against the bulk unprinted material. In this way, the present work assesses the viability of fused deposition 3D printing as a manufacturing process for the fabrication of high permittivity, complex shaped, devices.

## Results

**Figure 1:**
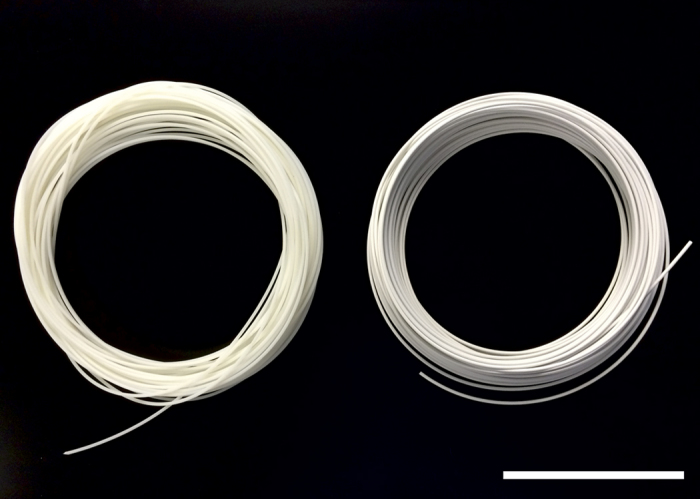
Coils of extruded filament, produced as feedstock for a fused deposition 3D printer. Left: unloaded ABS polymer. Right: BaTiO_3_/ABS polymer composite containing 50 wt% BaTiO_3_. Scale bar, 10 cm.

The BaTiO_3_ microparticles were dispersed in the ABS and extruded to form filament suitable for use as feedstock with a commercial desktop 3D printer (see Methods). A series of eight filaments were produced in lengths up to 220 m with loadings of 0 to 70 wt% BaTiO_3_ in ABS in 10 wt% increments. Loadings above 70 wt% were not investigated as the filament became too brittle to be readily printed. Lengths of unloaded (‘0 wt%’, ABS only) and loaded filament (50 wt% BaTiO_3_ in ABS) are shown in Fig. 1. To the naked eye, the ABS appears translucent off-white and becomes opaque and white with the addition of a substantial amount of BaTiO_3_ (though both materials are largely transparent to GHz microwaves).

**Figure 2:**
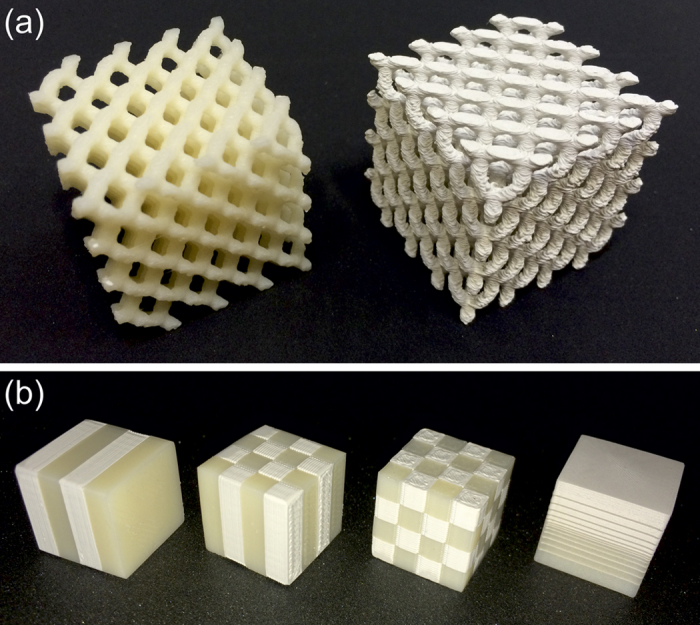
3D-printed polymer composite parts. (**a**) Example rod-connected diamond photonic crystal structures printed in ABS polymer (left, *ε*′ = 2.57) and 50 wt% BaTiO_3_/ABS polymer composite (right, *ε*′ = 4.95). Scale: each cubic structure has overall side length of 32 mm (8 mm unit cell). (**b**) Example 1D, 2D, and 3D periodic structures and a 1D graded structure printed using a combination of ABS polymer and 50 wt% BaTiO_3_ in ABS polymer composite. Scale: each cubic structure has side length of 16 mm.

Examples of printed objects are shown in Fig. 2, which explicitly confirm that the composite filament is readily compatible with a well-controlled printing process. The length scale of the macroscopic features of such prints are bounded above by the maximum build volume (i.e., the maximum volume of an object that may be printed by a given machine, which in this case was a cuboid of dimensions 24.6 cm × 15.2 cm × 15.5 cm, as determined by the size of the horizontal plate on which the print is deposited and the maximum vertical distance between the plate and the nozzles), and limited below by the layer resolution (in this case ≈0.1 mm). In between these length scales there is considerable scope for the production of microwave devices containing spatial variations in dielectric properties at arbitrary locations in 3D.

**Figure 3:**
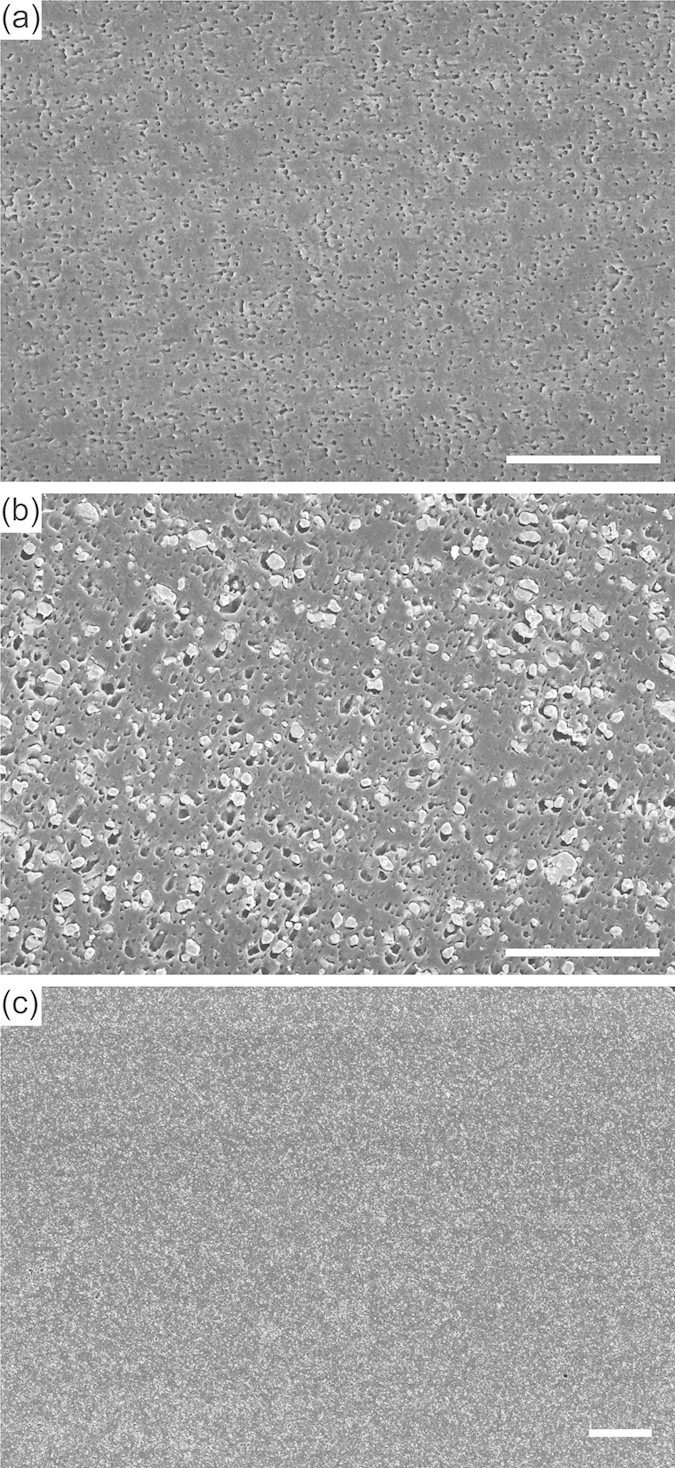
Scanning electron microscopy (conventional secondary electron imaging) of 3D printed parts. (**a**) Cross section of a part printed with unloaded ABS polymer (scale bar, 10 μm). (**b**) Cross section of a part printed with 50 wt% BaTiO_3_ in ABS polymer composite (scale bar, 10 μm). (**c**) A further, lower magnification, image of the 50 wt% printed part demonstrates that the particles are visibly well dispersed over length scales up to ~1 mm (scale bar, 50 μm).

At ~10 GHz microwave frequencies, the length scale of the microparticles is far below that of the operating wavelength and, despite being inhomogeneous on the length scale of a few microns, each feedstock material is dielectrically homogeneous and characterised, to very good approximation, by an effective averaged permittivity. Figure 3 shows typical scanning electron microscopy images of microtomed cross-sections of the 3D printed parts, comparing the polymer composite with the polymer only, and visibly demonstrating that the BaTiO_3_ microparticles were well-dispersed over length scales up to ~0.1 mm.

Using a printer with two nozzles, objects may be printed that are composed of two different feedstock materials, as shown in Fig. 2(b); in this case objects which incorporate both unloaded polymer and the 50 wt% polymer composite were printed. In the limit that the wavelength of electromagnetic radiation in the medium is much larger than the periodicity of such structures, they will behave as homogeneous media with an effective permittivity between that of the two feedstock materials. This effective permittivity may be tailored by modifying the relative thickness or volume fraction of each component. The 3D cubic structure shown in Fig. 2(b) will have an isotropic effective permittivity whereas the 1D and 2D periodic structures will be anisotropic. In general, such patterning enables the principal components of the effective permittivity tensor to be tuned. The graded test piece shown in Fig. 2(b) demonstrates the capability to print, for example, non-reflective boundaries. In the regime where the wavelength of the electromagnetic radiation is of the same order of magnitude as the periodicity of the structure, diffractive effects come into play and, if desired, may be exploited advantageously. In this regard the test pieces shown in Fig. 2 represent archetypal photonic crystal structures, which have applications in a wide range of devices including omnidirectional reflectors, filters, and waveguides^2^.

**Figure 4:**
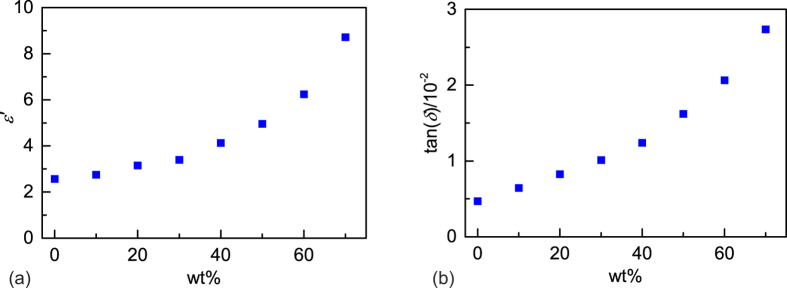
The real part of the permittivity and the loss tangent of 3D printed parts for various loading fractions of BaTiO_3_ in ABS. 1σ random uncertainties are within the thickness of the data symbols.

The bulk permittivity of the 3D-printed parts was determined, in the first instance, using a split-post dielectric resonator (SPDR) with a nominal measurement frequency of 15 GHz. The SPDR technique has been well-established to give accurate measurements of both *ε*′ and the loss tangent^29^,^30^. The loss tangent may be defined by tan(*δ*) = |*ε*″/*ε*′| where *ε*′ and *ε*″ are the real and imaginary parts of the dielectric permittivity respectively. The measurements were carried out on solid, disc-shaped test pieces of diameter ≈35 mm and thickness ≈0.5 mm. The results for parts printed from single filament-types with various loadings are listed in Table 1 and plotted in Fig. 4. The wt% values refer to the relative amounts of BaTiO_3_ and ABS in the composite as initially weighed out using a standard balance. The vol% values were inferred from the wt% values on the assumption of a binary mixture of BaTiO_3_ (density 6.08 g/cm^3^) and ABS (density 1.04 g/cm^3^) containing no air. Density measurements confirm that both the wt% and vol% values are accurate to within ≈1% for the final printed parts (see below). For each type of filament, six SPDR samples were printed and the quoted values represent the mean and standard uncertainty (1σ) on the mean of the six samples.

As expected, *ε*′ increased considerably as the fraction of BaTiO_3_ in the composite was increased, from 2.57 ± 0.02 for the unloaded polymer to 8.72 ± 0.04 for the 70 wt% polymer composite. tan(*δ*) also increased with increasing fraction of BaTiO_3_, from (0.469 ± 0.001) × 10^−2^ for the unloaded polymer to (2.736 ± 0.012) × 10^−2^ for the 70 wt% polymer composite.

The reproducibility of the entire process was investigated by fabricating a repeat batch of 20 wt% filament and testing six more printed samples with the SPDR. The mean values were *ε*′ = 3.13 ± 0.03 and tan(*δ*) = (0.846 ± 0.005) × 10^−2^, which may be compared against the 20 wt% values in Table 1, demonstrating that the permittivity values were reproducible over the entire process of feedstock powders to printed part, to within a few percent.

The SPDR technique provides values of *ε*′ and tan(*δ*) for a given sample at a single frequency: the resonant frequency of the device with the sample inserted. While the nominal frequency of the device was 15 GHz (the resonant frequency of the device with no sample present), the resonant frequency with the sample present depends on both the sample permittivity and its thickness. The frequency at which the results were obtained is indicated in the final column of Table 1, where the quoted value *f* represents the mean resonant frequency of the six samples at each loading fraction.

**Figure 5:**
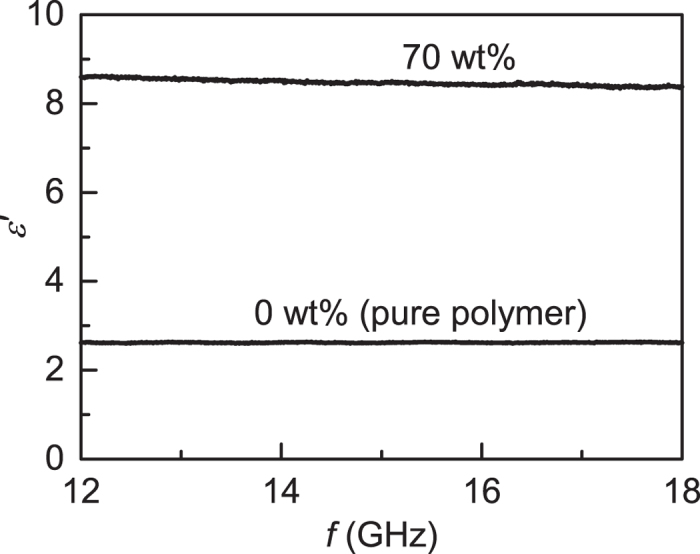
Real part of the permittivity *ε*′ as a function of frequency for ABS polymer only and 70 wt% BaTiO_3_/ABS polymer composite.

The frequency dispersion in the permittivity was investigated by carrying out additional measurements using a 7.9 mm × 15.8 mm rectangular waveguide with the Nicholson-Ross-Weir extraction method^31^,^32^. The 0 wt% and 70 wt% materials, being the extreme cases, were chosen for this test. The results are shown over the 12–18 GHz frequency range in Fig. 5. The samples exhibited relatively little dispersion over this range, with *ε*′ varying by less than 1.5%, even for the most highly loaded 70 wt% sample.

The waveguide technique also provided an independent method by which the SPDR measurements could be cross-checked. Four waveguide pieces were printed in unloaded ABS and tested. The values of *ε*′ were averaged over the range 12–18 GHz and averaged over the four samples, giving *ε*′ = 2.60 ± 0.01. This may be compared against *ε*′ = 2.57 ± 0.02 for the SPDR method, which suggested that the combined uncertainty in the SPDR measurements of *ε*′, including systematic effects, was ~1%.

The values of *ε*′ and tan(*δ*) for unloaded ABS were largely consistent with those reported in the literature, though a precise quantitative comparison was not possible due to expected differences in the properties of different grades of ABS. Riddle *et al.* reported *ε*′ = 2.73 ± 0.03 and tan(*δ*) = (0.75 ± 0.15) × 10^−2^ at 24 °C and ≈11 GHz for a sample of unprinted ABS of an unspecified grade or source (values extracted from Fig. 4 of ref. 27). Deffenbaugh *et al.*^28^ reported measurements on 3D printed parts for two different types of ABS at frequencies from 1 MHz to 11 GHz. At 11 GHz they reported *ε*′ = 2.54 (0.8%) and tan(*δ*) = 1.06 × 10^−2^ (5%) for ABS from the manufacturer Pure, and *ε*′_Lay_ = 2.6 (1.4%), *ε*′_St_ = 2.55 (2.5%), tan(*δ*)_Lay_ = 0.98 × 10^−2^ (96%), and tan(*δ*)_St_ = 1.30 × 10^−2^ (71%), for ABS-M30 manufactured by KeckFDM. Numbers in parentheses refer here to standard deviations of the sample sets, quoted as a percentage of the corresponding results. The multiple values for the latter material refer respectively to a “laying down orientation” and a “standing up” orientation of the print (i.e., the print had anisotropic dielectric properties; we carried out additional tests to confirm that no such anisotropy was present in our samples to within the overall accuracy level of 1%). Our value of *ε*′ = 2.57 ± 0.02 for pure printed ABS is consistent with those of Deffenbaugh *et al.*, whereas our value of tan(*δ*) = (0.496 ± 0.001) × 10^−2^ was approximately two times smaller than Deffenbaugh *et al.*’s, which may be attributable to the different grade of ABS used or to the large uncertainty in tan(*δ*) arising from the waveguide method employed in ref. 28.

*ε*′ and tan(*δ*) for the BaTiO_3_/ABS printed composites here may be compared with those measured for extruded, but unprinted, BaTiO_3_/ABS composite filament in ref. 16. However, a precise quantitative comparison is again limited due to the expected differences in the properties of different grades of ABS and, further, expected differences in the properties of different ‘grades’ of BaTiO_3_ particles. At 15 vol%, Moulart *et al.*^16^ reported *ε*′ = 6.77 and tan(*δ*) = 2.1 × 10^−2^, compared with *ε*′ = 4.95 ± 0.01 and tan(*δ*) = (1.622 ± 0.001) × 10^−2^, and at 30 vol% *ε*′ = 8.73 and tan(*δ*) = 3.3 × 10^−2^, compared with *ε*′ = 8.72 ± 0.04 and tan(*δ*) = (2.736 ± 0.012) × 10^−2^ at 29 vol%.

The dielectric properties of the printed parts depend to a certain extent on the ‘quality’ of the filament and the control over the printing process. In particular, *ε*′ will be reduced if any voids are trapped within the print. Residual acetone—which was used as a solvent in the production of the composite filament (Methods)—could cause bubbling of the filament and hence reduced filament density when it was extruded. This detrimental effect was practically eliminated using an extended powder drying stage to ensure essentially complete solvent evaporation prior to extrusion. To quantify this, density measurements were carried out using a microbalance via the buoyancy method. The 40 wt% composite was arbitrarily chosen for comparative studies. Bulk unprinted samples were prepared by hot-pressing pieces of the filament feedstock into a solid block, followed by cutting and grinding to shape. The bulk density of the unloaded, unprinted, ABS was measured to be 1.04 ± 0.01 g/cm^3^. Using this value together with the manufacturer’s value for the particle density of the BaTiO_3_ microparticles of 6.08 g/cm^3^, the expected value for the density of a binary mixture (no air) of 40 wt% BaTiO_3_ in ABS is 1.556 g/cm^3^. The actual bulk density of the 40 wt% composite, averaged over four samples, was measured to be 1.553 ± 0.002 g/cm^3^. Thus, the density measurements revealed that, with appropriate drying, the composite feedstock filament density matched that expected from a binary mixture of ABS and BaTiO_3_ to within ≈0.2%.

**Figure 6:**
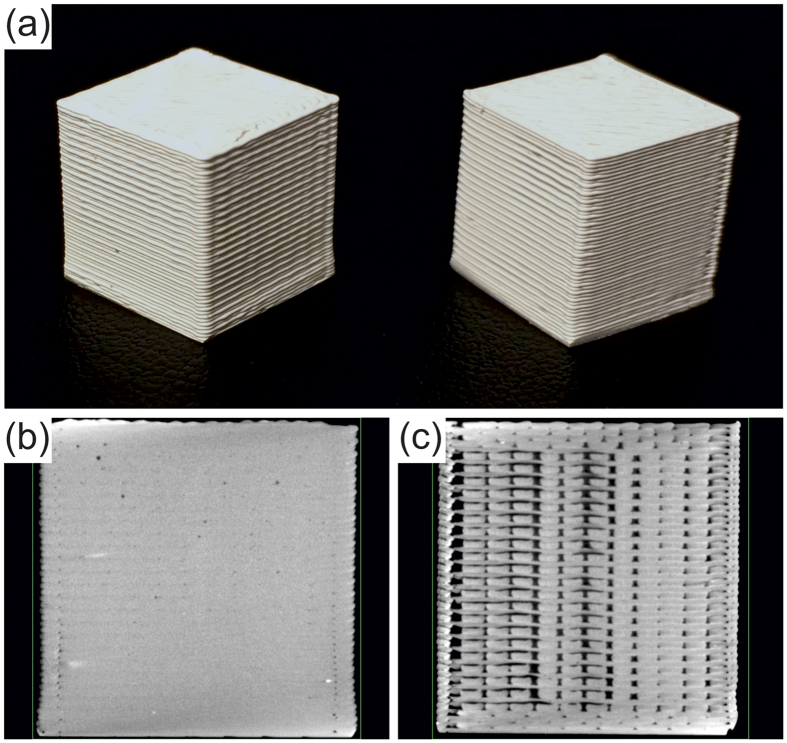
Comparison of ‘good’ and ‘bad’ quality prints using 50 wt% BaTiO_3_/ABS polymer composite. (**a**) A photograph of a typical good print (left) and a bad print (right) indicates that, externally, the prints appear largely similar. (**b**) A typical X-ray tomography image shows relatively little voiding in the good quality sample, whereas a corresponding typical image for the bad quality sample shows a relatively high level of voiding. The tomography slices show planes that are vertical with respect to the orientation shown in the photograph. The bad quality print is shown for illustration only, and all other data reported in this Article concern good prints of the quality shown in (**b**). Scale: cubes are 1 cm^3^.

The printing process itself can introduce voids, even if a structure with 100% infill is intended. This is shown in Fig. 6, where a ‘good’ quality print is compared against a ‘bad’ quality print. Externally, the prints appear largely similar, as shown in Fig. 6(a). However, X-ray computed tomography images revealed a significant difference in the internal void content, as shown in Fig. 6(b,c). Voids will reduce the overall effective permittivity. The bad quality print shown in Fig. (6) was generated specifically for the purpose of this illustration by reducing the feed rate of the filament during printing below its optimum value; all other data reported in this article concerns good quality prints of the type shown in Fig. 6(b). To put a quantitative limit on the total amount of voiding in such prints (which is the relevant parameter with respect to the permittivity measurements), we compared the density of typical 40 wt% composite printed parts against the above density of the unprinted bulk composite. The density of the equivalent printed samples, again averaged over four samples, was measured to be 1.544 ± 0.008 g/cm^3^. Thus, there was no significant difference between the bulk density of the printed parts and the bulk density of the unprinted material when working to an overall uncertainty of ~1%. As a result, the volume percentages quoted in Table 1, which were calculated from the measured wt% on the assumption of no air, are valid to within ~1% uncertainty. Each set of four printed and unprinted bulk samples were measured using the SPDR and the mean values of *ε*′ were found to be 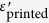 = 4.13 ± 0.01, and 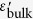 = 4.09 ± 0.03. Thus, there is no statistically significant difference in the values of *ε*′ between the printed and unprinted bulk samples at this level of uncertainly.

## Discussion

The above results confirm that, using polymer composite feedstock that can be processed in long lengths, an inexpensive commercial desktop 3D printer can be used to fabricate geometrically complex parts with tailored, and relatively high (compared to conventional polymers), real permittivity at microwave frequencies. Importantly, the dielectric properties of the 3D printed parts can be the same, to within 1% uncertainty, as bulk unprinted materials. We suggest the results confirm that 3D printing of even heavily loaded composite filament can be considered sufficiently reproducible to be deployed as a manufacturing process.

A limit to this approach is the fraction of ceramic particles that can be added to the polymer matrix before the filament becomes too brittle to be compatible with the fused deposition printing process. Accordingly, the maximum fraction used in this study was 70 wt% (29 vol%) of BaTiO_3_. However, there would appear to be considerable room for improvement in this regard by appropriate choice of the type and grade of polymer and the addition of additives such as a plasticiser. For example, McNulty *et al.*^33^ investigated binder formulation in the context of the ‘fused deposition of ceramics’ technique^11^,^12^ and achieved ceramic filling fractions of >50 vol%. It should be noted that alternative 3D printing processes such as ceramic stereolithography^6^,^7^ can produce parts made from high performance dielectric materials without this limitation^8^,^9^,^10^, and can typically achieve higher resolution than fused deposition. The primary advantages of the fused deposition method are that the machines can be relatively inexpensive, and that they can readily print composites based on a wide range of common thermoplastics (ABS, polyethylene terephthalate, nylon, polypropylene, etc.) While the exact composition of the polymer composite will be dictated by the combination of properties required for a given application (electromagnetic, mechanical, fire retardance, chemical resistance, etc.), the detailed dielectric characterisation of the archetypal binary BaTiO_3_/ABS system described here demonstrates the robustness and reproducibility of the general method, indicating that it could potentially be exploited in the manufacture of a wide range of electromagnetic devices.

## Methods

The BaTiO_3_ microparticles (nominal particle size specified by the manufacturer as <3 μm, Sigma-Aldrich, St. Louis) were dispersed in the ABS (MFI-22, Styrolution, Frankfurt am Main) as follows. First, the ABS pellets were dissolved in acetone, typically using about one litre of acetone per 200 g of ABS. This created a solution with sufficiently low viscosity that the BaTiO_3_ microparticles could be readily added and mechanically mixed in by hand. The resulting suspension was then left at room temperature in an open container and mixed periodically; the viscosity increased as the acetone evaporated off, resulting in reduced sedimentation. With the suspension in a high-viscosity and well dispersed state, the material was spread on polytetrafluroethylene trays and left to solidify via continued evaporation of acetone. The resulting sheets of polymer composite were then ground using a kitchen blender into a coarse powder. The powder was then left, typically for about a week, in a drying oven at ≈70 °C, to ensure effective removal of any remaining acetone.

The composite powders were extruded using a Noztek Pro filament extruder (Noztek, London) to form filament suitable for use as feedstock in fused deposition 3D printers. With appropriate choice of the nozzle diameter (in the range 1.4–1.7 mm) and careful control of the nozzle temperature (typically in the range 190–210 °C), filaments with a circular cross section of average diameter of 1.75 mm and a typical standard deviation of 0.02–0.03 mm were produced.

Objects were printed using a Makerbot Replicator 2X desktop 3D printer (Makerbot Industries, New York City) with standard settings: e.g., 230 °C nozzle temperatures and 110 °C printed bed temperature. A 15 GHz SPDR (QWED, Warsaw) and a Ku band waveguide were used in conjunction with a standard vector network analyzer to measure the dielectric properties at microwave frequencies. Since extraction of the permittivity using the SPDR and waveguide techniques assume perfectly flat samples, yet the fused deposition printing produces objects with a potentially significant degree of non-flat surface topology, all samples were printed over-sized and subsequently ground down. The thickness of a given sample, which is required for extraction of the permittivity, was measured using a micrometer screw gauge.

Cross-sections for electron microscopy analysis were prepared using an Ultracut E, ultramicrotome (Reichert-Jung Inc., Austria) with a diamond blade, before sputter-coating with ≈1 nm Pt. Conventional secondary electron imaging was carried out using a JEOL JSM-840F electron microscope (JEOL USA, Inc., Peabody, USA).

All quoted permittivity values in the main text pertain to measurements conducted on 3D printed parts, except the 70 wt% waveguide results plotted in Fig. 5. It was found that waveguide measurements on highly-loaded materials with relatively large *ε*′ were prone to error due to small gaps between the sample and the walls of the waveguide. To mitigate against this source of error, the 70 wt% filament was hot-pressed directly into the waveguide test piece to create an almost perfect seal between the sample and the waveguide walls. The primary purpose of the 70 wt% waveguide measurement was to investigate the approximate level of dispersion, not to provide a particularly accurate value *ε*′ for the 70 wt% composite. All permittivity measurements were carried out at a room temperature of 21–22 °C.

Cone beam X-ray computed tomography was performed by using an ImagiX CT system (North Star Imaging Inc., Minnesota, USA). The scanning conditions were as follows: X-ray tube with a tungsten target, 140 kV tube voltage, 50 μA tube current, and frame rate of 2 Hz. The isotropic voxel size was 16.2 μm. The projection data was then reconstructed and visualised using efX-CT software (version 1.7.397.29, North Star Imaging Inc., Minnesota, USA).

## Additional Information

**How to cite this article**: Castles, F. *et al.* Microwave dielectric characterisation of 3D-printed BaTiO_3_/ABS polymer composites. *Sci. Rep.* **6**, 22714; doi: 10.1038/srep22714 (2016).

## Figures and Tables

**Table 1:** Dielectric characterisation of 3D-printed polymer composite parts with various loadings of BaTiO_3_ in ABS.

wt% BaTiO_3_	vol% BaTiO_3_	*ε*′	tan(*δ*)/10^−2^	*f* (GHz)
0	0	2.57 ± 0.02	0.469 ± 0.001	14.83
10	1.9	2.75 ± 0.02	0.643 ± 0.003	14.82
20	4.1	3.15 ± 0.01	0.827 ± 0.003	14.74
30	6.8	3.40 ± 0.01	1.012 ± 0.004	14.74
40	10	4.13 ± 0.01	1.240 ± 0.005	14.63
50	15	4.95 ± 0.01	1.622 ± 0.001	14.55
60	20	6.24 ± 0.03	2.064 ± 0.005	14.42
70	29	8.72 ± 0.04	2.736 ± 0.012	14.13
